# Hawthorn Fruit Extract Elevates Expression of Nrf2/HO-1 and Improves Lipid Profiles in Ovariectomized Rats

**DOI:** 10.3390/nu8050283

**Published:** 2016-05-13

**Authors:** Jeong-Hyun Yoo, Yanan Liu, Hyun-Sook Kim

**Affiliations:** 1 Division of Food and Nutritional Science and Life Systems, College of Science, Sookmyung Women’s University, Seoul 04310, Korea; yjh8252@naver.com; 2 Major in Food and Nutrition, College of Human Ecology, Sookmyung Women’s University, Seoul 04310, Korea; smileyanan@naver.com

**Keywords:** hawthorn extract, antioxidant, oxidative stress, nuclear factor erythroid 2 related factor, heme oxygenase-1, glutathione peroxidase, ovariectomized

## Abstract

The purpose of this study was to investigate the effects of hawthorn (*Crataegus pinnatifida* Bunge) extract on the lipid profiles and antioxidant properties in ovariectomized (OVX) rats. After ovariectomy, the rats were randomly divided into four groups: the non-OVX control (Sham), the OVX-control (OVX), the OVX + 100 mg/kg b.w. of hawthorn extract (OL), and the OVX + 200 mg/kg b.w. of hawthorn extract (OH). The final body weights of the OVX group were significantly increased, but the increment was significantly decreased in hawthorn groups (*p* < 0.05). The serum total and low-density lipoprotein (LDL) cholesterol levels were significantly elevated in the OVX group, whereas the hawthorn groups showed a significant decrease in these levels (*p* < 0.05). The hepatic triglyceride (TG) and malondialdehyde (MDA) levels were significantly reduced in the hawthorn groups compared with the OVX group (*p* < 0.05). The mRNA expression of nuclear factor erythroid 2–related factor (Nrf2), heme oxygenase-1 (HO-1), and glutathione peroxidase (GPx) were significantly decreased in the OVX group, whereas the hawthorn groups exhibited a significant increase in expression (*p* < 0.05). The protein expressions of Nrf2, HO-1, and GPx were lower in the OVX group than the Sham group (*p* < 0.05). The oral administration of hawthorn extract reversed the suppression of protein levels. These results suggest that hawthorn extract could have protective effects in OVX rats by improving lipid profiles, decreasing oxidative stress, and improving the antioxidant defense system.

## 1. Introduction

Metabolic disorders occurring in menopausal women, including dyslipidemia, hypertension, type 2 diabetes, endothelial dysfunction, and vascular inflammation, constitute risk factors for cardiovascular disease [1,2]. The lipid and lipoprotein metabolism is markedly altered in postmenopausal women when elevated total cholesterol, low-density lipoprotein (LDL) cholesterol, and very low-density lipoprotein (VLDL) cholesterol have been observed [3]. Clinical epidemiological studies have shown that postmenopausal women and ovariectomized patients have an increased risk of cardiovascular diseases, partially because of estrogen deficiency–induced oxidative stress. Furthermore, oxidative stress may aggravate or accelerate established cardiovascular diseases [4].

Oxidative stress is a biochemical disequilibrium propitiated by the excessive production of free radicals and reactive oxygen species (ROS). Antioxidant enzymes, such as catalase (CAT), superoxide dismutase (SOD), and glutathione peroxidase (GPx), play an important role in protection of the body from oxidative damage by ROS [5]. Nuclear factor erythroid 2–related factor (Nrf2) protects cells against oxidative stress through the antioxidant response element (ARE)-mediated induction of several phase 2 detoxifying and antioxidant enzymes, such as heme oxygenase-1 (HO-1), SOD, GPx, CAT, and glutathione S-transferase (GST) [6,7]. Among all these cytoprotective enzymes, HO-1 plays a crucial role in protecting cells against oxidative stress [8]. Moreover, recent data indicates that estrogen deficiency in the menopausal state leads to an increase in oxidative stress and ovariectomy induces diminished HO activity and expression [9]. Procyanidins, quercetin, resveratrol, and curcumin [10], as well as other flavonoids or polyphenols, have been reported to up-regulate HO-1 expression by activating Nrf2 to bind with ARE in the HO-1 gene promoter region [11,12]. 

Hawthorn (*Crataegi fructus*), also known as haw, maybush, or whitehorn, is part of a genus of spiny shrubs and trees and has been widely used for foodstuffs and traditional medicine in Europe, Asia, and North America [13]. There are many different species of hawthorn, which is cultivated in various countries [14]. Particularly, *Crataegus pinnatifida* Bunge is widely distributed in Asia (Korean, China and Japan) [15,16]. The main biologically active components reported in hawthorn are B-type procyanidins, epicatechin, chlorogenic acid, hyperoside, procyanidin C1, and rutin [17]. According to the results of previous studies, procyanidin content is higher in hawthorn than in other fruit sources, such as grape, apple, red raspberry, and strawberry [18]. The antioxidant activity of hawthorn has been reported to scavenge free radicals [19], and prevent lipid peroxidation [20]. It has also been reported that hawthorn is effective for atherosclerosis [21], cardiovascular protection [22], endothelium-dependent vasorelaxation [23], and coronary circulation [24].

The aim of this study was to investigate the effect of hawthorn fruit extract on the lipid profiles and antioxidant properties in ovariectomized (OVX) rats. We hypothesized that hawthorn extract administration would regulate the hepatic expression of genes related to the Nrf2/HO-1–mediated pathway.

## 2. Materials and Methods 

### 2.1. Preparation of Hawthorn Fruit Extract

Dried hawthorn powder (whole fruits) was purchased from herbal medicine store (Seoul, Korea). The hawthorn (*Crataegus pinnatifida* Bunge) used to make dried powder were planted at Jeonnam, Korea. A total of 50 g of hawthorn powder was refluxed with 500 mL of 70% ethanol at 63 °C for four hours. The mixture was centrifuged at 3000 rpm for 30 min and filtered using filter paper (3MM CHR, Whatman, Maidstone, UK), and then the filtrates were concentrated using a rotary evaporator (N-21NS; EYELA, Tokyo, Japan) at 40 °C. The resulting filtrates were dried using a freeze drier (Operon FDUT-8606, Gimpo, Gyeonggi, Korea) and then stored at −18 °C until further analysis. The yield of the hawthorn extraction was 25.07%.

### 2.2. Animals and Diets 

Nine-week-old female Sprague-Dawley rats (KOATECH, Gyeonggi-do, Korea) were housed in controlled temperature (21 ± 1 °C) and humidity (50%–60%) conditions in a 12-h light/12-h dark cycle with free access to water and an AIN-76A diet (Research diet, New Brunswick, NJ, USA). After acclimation for two weeks, the animals were either OVX or sham-operated. The surgical procedure was performed under general anesthesia and administered as an intraperitoneal injection of 0.2–0.23 mL with a combination of Zoletil and Rompun in a 1:1 ratio. A bilateral (left and right) incision (1–2 cm) that included the skin, muscle, and peritoneum was performed 2 cm below the last rib, and the left and right ovaries were extirpated. For the sham operation, only a small amount of fat around each ovary was extirpated. After two weeks of recovery, the rats were randomly divided into four groups (*n* = 7 for each group): the non-OVX control (Sham), the OVX-control (OVX), the OVX + 100 mg/kg b.w. of hawthorn extract (OL), and the OVX + 200 mg/kg b.w. of hawthorn extract (OH). All the animal procedures were performed in accordance with the guidelines issued by the Animal Ethical Committee of Sookmyung Women’s University for the care and use of laboratory animals (SMWU-IACUC-1502-023). The rats in the treatment group were orally administered hawthorn extract, which was dissolved in distilled water every day for eight weeks. The control groups were administered 1 mL of distilled water. Food intakes were measured every other day, and body weights were monitored once a week. The food efficiency ratio (FER) was calculated by following formula (1).

Food efficiency ratio (FER%) = Body weight gain (g)/food intake (g/day)
(1)

### 2.3. Sample Preparation

After an overnight fast, the final body weights were measured and the rats were euthanized using CO_2_. The blood was collected by cardiocentesis to determine the serum lipid profiles. The serum was separated by centrifugation at 3000 rpm for 30 min (Combi-514R, Hanil Co. Ltd., Seoul, Korea). The serum and livers were stored at −70 °C until analysis (DF8517; Ilshin Laboratory Co., Ltd., Seoul, Korea).

### 2.4. Determination of Blood Biochemical Variables

Serum estradiol was measured using the Estradiol kit (ES180S-100, CalBiotech, Spring Valley, CA, USA). Serum triglyceride (TG), total cholesterol, and high-density lipoprotein (HDL) cholesterol levels were determined using a Cleantech TG-S kit (3I1570; Asanpharm, Hwaseong, Korea), T-CHO kit (3I2020; Asanpharm, Hwaseong, Korea), and HDL-CHO (3I2030; Asanpharm, Hwaseong, Korea). Serum free fatty acid (FFA) and GPx were measured by commercially available kits using an FFA assay kit (EFFA-100, BioAssay Systems, Hayward, CA, USA) and GPx activity colorimetric assay kit (K762-100, BioVision, CA, USA) with an Epoch microplate spectrophotometer (BIOTEK, Inc., Winooski, VT, USA). 

Serum CAT activity was assayed following the method of Aebi [25]. In brief, 1.9 mL of the phosphate buffer (50 mM, pH 7.0) and 250 μL per sample were added to a 15 mL Falcon tube, and the reaction was started by the addition of 1 mL of 30 mM hydrogen peroxide (H_2_O_2_). The decomposition of H_2_O_2_ was monitored at 240 nm at 25 °C. One unit of CAT activity was defined as an absorbance change of 0.01 as units/min [26].

Serum LDL cholesterol (2), VLDL cholesterol levels (3), non-HDL cholesterol (4), and Atherogenic Index (AI, (5)) were calculated using the following formula [27]:

LDL cholesterol (mg/dL) = total cholesterol level − (HDL cholesterol level + TG level/5)
(2)

VLDL cholesterol (mg/dL) = TG level/5
(3)

non-HDL cholesterol = total cholesterol level − HDL cholesterol level
(4)

AI = (total cholesterol level − HDL cholesterol level)/HDL cholesterol level
(5)

### 2.5. Liver Analysis

The extraction was a modification of the Folch method [28]. Approximately 0.1 g of frozen liver was minced and transferred into a test tube. A total of 3 mL of chloroform/methanol (2:1, v/v) was then added and followed by a 2 min homogenization. The suspension was vortexed at room temperature for 20 min. After vortexing, 2 mL of double-distilled water was added. The samples were then centrifuged at 3000 rpm for 20 min. After centrifugation, the bottom layer was carefully aspirated into a new test tube and wash dried by stream of nitrogen. The dried lipid was weighed and re-dissolved in methanol and used for lipid analysis. The liver TG content was determined using the TG-S kit (3I1570; Asanpharm, Hwaseong, Korea).

The determination of malondialdehyde (MDA) by thiobarbituric acid (TBA) was used as an index of the extent of lipid peroxidation [29]. Briefly, 0.3 g of liver tissue was homogenated with 3 mL of cold 10% trichloroacetic acid (TCA). The homogenate was centrifuged at 3000 rpm for 30 min at RT. The supernatant was mixed with 2 mL of 0.6% TBA and placed in a hot water bath for 15 min and then cooled. The absorbance of clear supernatant was measured at 450, 532, and 600 nm. The results were expressed as MDA formation per milligram of protein. The MDA content was calculated using the following formula:

MDA content (μmol·L^−1^) = 6.45 × (A_532_ − A_600_) − 0.56 × A_450_ (6)

### 2.6. mRNA Expression Analysis

RNA was isolated from homogenized liver using an easy-spin™ total RNA extraction kit (17221, iNtRON Biotechnology, Gyeonggi-do, Korea). The reverse transcription–polymerase chain reaction (RT-PCR) was performed in a PCR machine (Bio-Rad Laboratories, Hercules, CA, USA) using a Maxim RT-PCR Premix kit (25131, iNtRON Biotechnology, Gyeonggi-do, Korea). Details of the primers and optimal cycling conditions are shown in Table 1. The PCR products were visualized after electrophoresis in 2% agarose gel. All gene expressions were normalized to Glyceraldehyde-3-phosphate dehydrogenase (GAPDH), which served as an internal control for the quality of isolated RNA from each homogenized liver sample.

### 2.7. Western Blot Analysis

The total protein was extracted from 5 mg of liver tissue using a Pro-prep kit (17081, iNtRON Biotechnology, Gyeonggi-do, Korea), and the protein concentration of the samples was determined using the PRO-MEASURE™ kit (17081, iNtRON Biotechnology, Gyeonggi-do, Korea). The samples (50 μg each) were separated by denaturing SDS-PAGE and collected on a PVDF membrane (Merck Millipore, Bedford, MA, USA) by electrophoretic transfer (Bio-Rad Laboratories, Inc., Hercules, CA, USA). The membrane was pre-blocked with 5% skim milk in PBS solution containing 0.1% Tween-20 (PBST) for one hour and incubated overnight with the primary antibody: Nrf2 (C-20) polyclonal antibody (1:200 dilution; Santa Cruz Biotechnology Inc., Paso Robles, CA, USA), HMOX1 polyclonal antibody (1:1000 dilution; Abnova, Taipei, Taiwan), and Gpx1 polyclonal antibody (1:500 dilution; Abnova, Taipei, Taiwan). Each membrane was washed three times for 10 min and incubated with Goat Anti-Rabbit IgG H&L (HRP) secondary antibody (1:7500 dilution Abnova, Taipei, Taiwan) and Donkey-Anti-Goat IgG H&L (HRP) secondary antibody (1:7500 dilution Abnova, Taipei, Taiwan). To correlate protein loading, the blots were analyzed for GAPDH expression using a GAPDH polyclonal antibody (1:1000 dilution; Abnova, Taipei, Taiwan). Chemiluminescence was detected with the Immobilon western horseradish peroxidase substrate (Merck Millipore, Bedford, MA, USA). The intensity of the immunoreactive bands was determined by densitometric analysis (LAS-3000, Fujifilm Co., Ltd., Tokyo, Japan).

### 2.8. Statistical Analysis

Statistical analysis was performed using SAS 9.4 (SAS Institute Inc., Cary, NC, USA). All data are presented as mean ± SD. The results from each experimental group were compared using one-way ANOVA. Differences in mean values among the four groups were tested using Duncan’s multiple tests. A *p* value of < 0.05 was considered statistically significant.

## 3. Results

### 3.1. Food Intake, Food Efficiency Ratio, Body Weight, and Organ Weight

**Figure 1:**
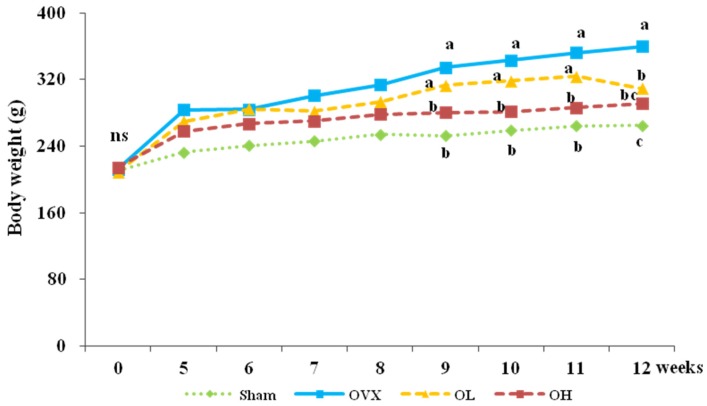
Body weight change of each group. Values are mean ± SD (*n* = 7 for each group). The different letters (a > b > c) within a column indicate significant differences (*p* < 0.05) determined by Duncan’s multiple range test; Sham, the non-OVX control; OVX, the OVX-control; OL, the OVX + 100 mg/kg b.w. of hawthorn extract; OH, the OVX + 200 mg/kg b.w. of hawthorn extract.

The food intake, food efficiency ratio (FER), body weight, and organ weight are presented in Table 2. No differences in food intakes were observed among groups throughout the eight-week feeding period. The FERs were significantly higher in the OVX group than in the Sham group (*p* < 0.0001). Compared with the OVX group, the OL and OH groups had significantly decreased FERs (*p* = 0.0498, *p* = 0.001). The initial body weights were not significantly different among all groups. After ovariectomy, a significant increase in body weights was observed in the OVX group compared with the Sham group. The administration of a high dose of hawthorn extract significantly lowered body weights from week 5 until the end of the experimental period. The final body weights were also significantly lower in the OL group compared with the OVX group (Figure 1). The OVX group had the highest liver weights (*p* = 0.0029), and the OL and OH groups had liver weights similar to those of the Sham group (*p* = 0.0407, *p* = 0.0103). Compared with the Sham group, the OVX group showed significantly decreased uterine weights (*p* < 0.0001) and increased uterine fat weights (*p* = 0.0014). The administration of a high dose of hawthorn extract significantly increased the uterine weights (*p* < 0.05), and the consumption of any dose of hawthorn extract lowered the uterine fat weights (*p* < 0.05).

### 3.2. Serum Estradiol Level

**Figure 2:**
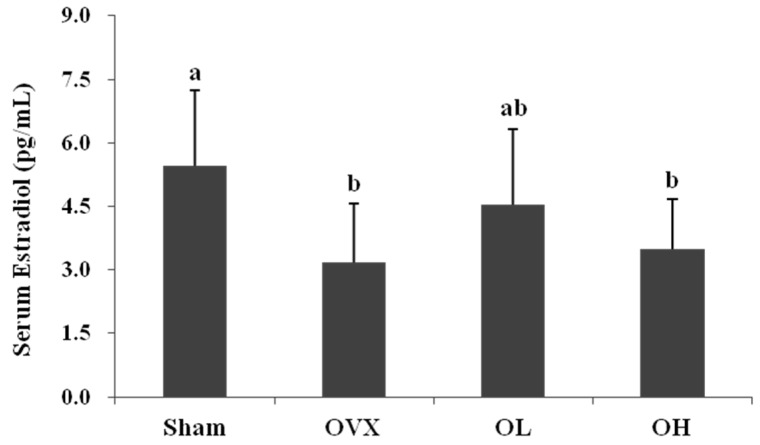
Serum estradiol level of each group. Values are mean ± SD (*n* = 7 for each group); the different letters (a > b > c) within a column indicate significant differences (*p* < 0.05) determined by Duncan’s multiple range test; Sham, the non-OVX control; OVX, the OVX-control; OL, the OVX + 100 mg/kg b.w. of hawthorn extract; OH, the OVX + 200 mg/kg b.w. of hawthorn extract.

The serum estradiol levels were significantly higher in the Sham group than in the OVX group (*p* = 0.0186), but the levels were not significantly different between the OVX group and the hawthorn extract groups (Figure 2).

### 3.3. Serum Lipid Profiles

**Figure 3:**
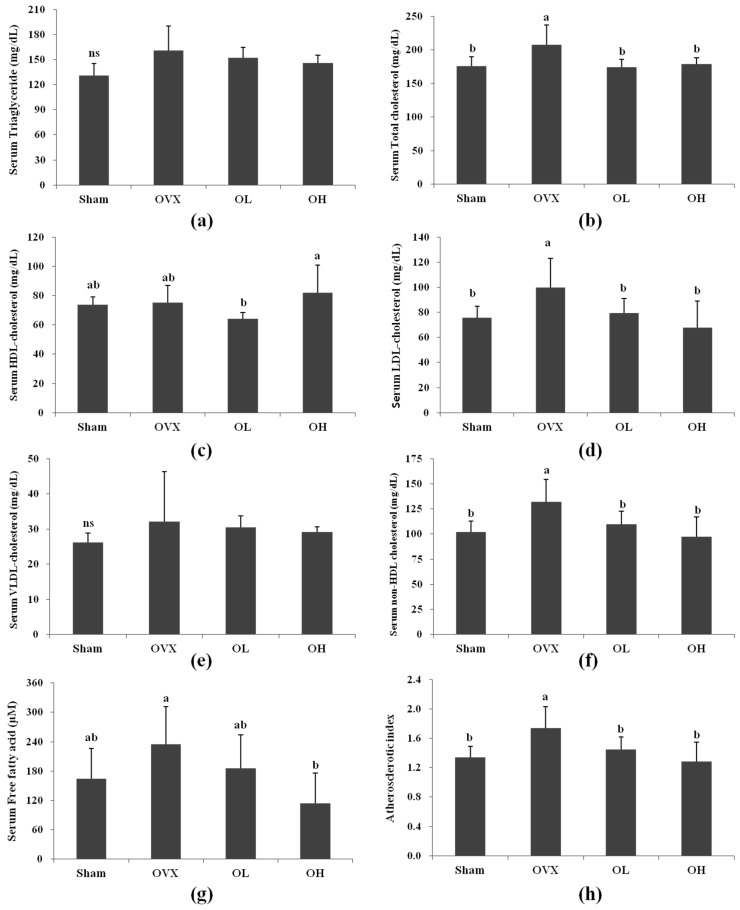
Blood biochemical variables of each group. (**a**) Serum triglyceride; (**b**) Serum total cholesterol; (**c**) Serum HDL cholesterol; (**d**) Serum LDL cholesterol; (**e**) Serum VLDL cholesterol; (**f**) Serum non-HDL cholesterol; (**g**) Serum free fatty acid; (**h**) Atherosclerotic index. ns, not significant; Values are mean ± SD (*n* = 7 for each group). The different letters (a > b > c) within a column indicate significant differences (*p* < 0.05) determined by Duncan’s multiple range test; Sham, the non-OVX control; OVX, the OVX-control; OL, the OVX + 100 mg/kg b.w. of hawthorn extract; OH, the OVX + 200 mg/kg b.w. of hawthorn extract;

The serum lipid profiles are shown in Figure 3. In the OVX group, the total cholesterol and LDL cholesterol levels increased 18% and 32%, respectively, compared with those of the Sham group. The total cholesterol levels were 16% and 14% lower in the OL and OH groups than that of the OVX group, which was similar to the total cholesterol level in the Sham group. The LDL cholesterol levels were lower in the OL and OH groups (20% and 32%, respectively) compared with the OVX group. Compared with the OVX group, the OH group showed a 51% decrease in FFA, while there was no difference in the FFA levels between the OVX and Sham groups. No significant changes were observed in the serum TG and VLDL cholesterol levels among all groups. There was no difference in serum HDL cholesterol levels between the OVX and the Sham groups. The non-HDL cholesterol levels were higher in the OVX group than in the Sham group (30%). After eight weeks of administration, the OL and OH groups had significantly lower non-HDL cholesterol levels than the rats in the OVX group by 17% and 26%, respectively. The OVX group showed a marked increase in AI compared with the Sham group (*p* = 0.0076), whereas those of the OL and OH groups were significantly lower compared with the OVX group (*p* = 0.0422 and *p* = 0.0095, respectively).

### 3.4. Serum Antioxidant Enzyme Activities

**Figure 4:**
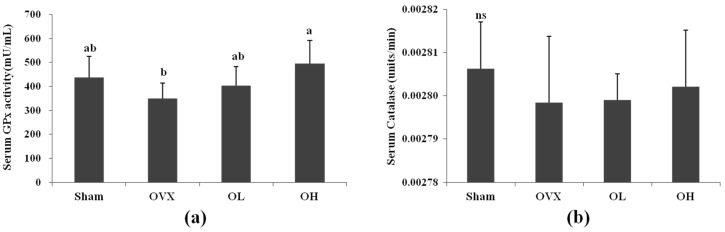
Serum antioxidant enzyme activities of each group. (**a**) Serum GPx; (**b**) Serum catalase. ns, not significant; Values are mean ± SD (*n* = 7 for each group). The different letters (a > b > c) within a column indicate significant differences (*p* < 0.05) determined by Duncan’s multiple range test; Sham, the non-OVX control; OVX, the OVX-control; OL, the OVX + 100 mg/kg b.w. of hawthorn extract; OH, the OVX + 200 mg/kg b.w. of hawthorn extract; GPx, Glutathione peroxidase.

Figure 4 shows the effect of hawthorn extract on the antioxidant activity in serum. Serum GPx activity showed a 42% increase in the OH group compared with the OVX group. No significant changes in CAT activity were observed among all groups.

### 3.5. Liver TG and MDA Levels

**Figure 5:**
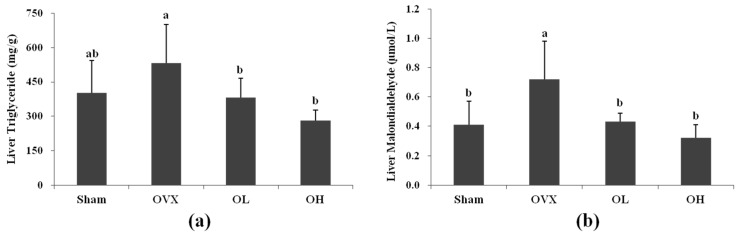
Liver triglyceride and malondialdehyde of each group. (**a**) Liver triglyceride; (**b**) Liver malondialdehyde. Values are mean ± SD (*n* = 7 for each group). The different letters (a > b > c) within a column indicate significant differences (*p* < 0.05) determined by Duncan’s multiple range test; Sham, the non-OVX control; OVX, the OVX-control; OL, the OVX + 100 mg/kg b.w. of hawthorn extract; OH, the OVX + 200 mg/kg b.w. of hawthorn extract.

The hepatic TG levels of the OL and OH group were 28% and 47% lower than that of the OVX group, respectively, while there were no differences in the hepatic triglyceride level between the OVX and Sham groups. The MDA level in the OVX group increased by 75% compared with that of the Sham group, while the OL and OH groups showed a 40% and 55% reduction in MDA level, respectively (Figure 5).

### 3.6. mRNA Expression of Nrf2, HO-1, GPx, and CAT in Liver

**Figure 6:**
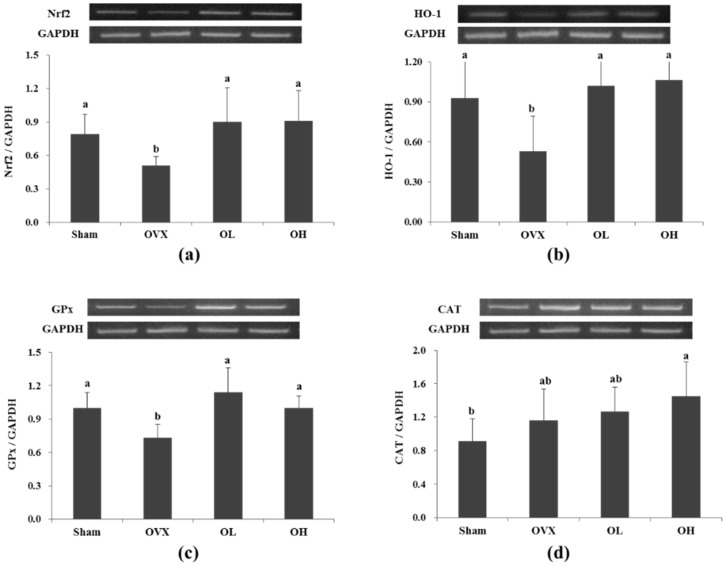
mRNA expression of Nrf2, HO-1, GPx and CAT in liver of each group. (**a**) Nrf2; (**b**) HO-1; (**c**) GPx; (**d**) CAT. Values are mean ± SD (*n* = 7 for each group). The different letters (a > b > c) within a column indicate significant differences (*p* < 0.05) determined by Duncan’s multiple range test; Sham, the non-OVX control; OVX, the OVX-control; OL, the OVX + 100 mg/kg b.w. of hawthorn extract; OH, the OVX + 200 mg/kg b.w. of hawthorn extract; Nrf2, Nuclear factor erythroid 2–related factor; HO-1, Heme oxygenase-1; GPx, Glutathione peroxidase; CAT, Catalase; GAPDH, Glyceraldehyde-3-phosphate dehydrogenase.

The mRNA expression of Nrf2, HO-1, and GPx was significantly decreased in the OVX group compared with the Sham group (*p* = 0.003, *p* = 0.023, and *p* = 0.002, respectively); however, the rats in the hawthorn groups exhibited a significant increase in expression of Nrf2 (*p* = 0.007 and *p* = 0.007, respectively), HO-1 (*p* = 0.008 and *p* = 0.006, respectively), and GPx (*p* = 0.001 and *p* = 0.001, respectively). The mRNA expression of CAT was not significantly different among all the groups (Figure 6).

### 3.7. Protein Expression of Nrf2, HO-1, and GPx in Liver

**Figure 7:**
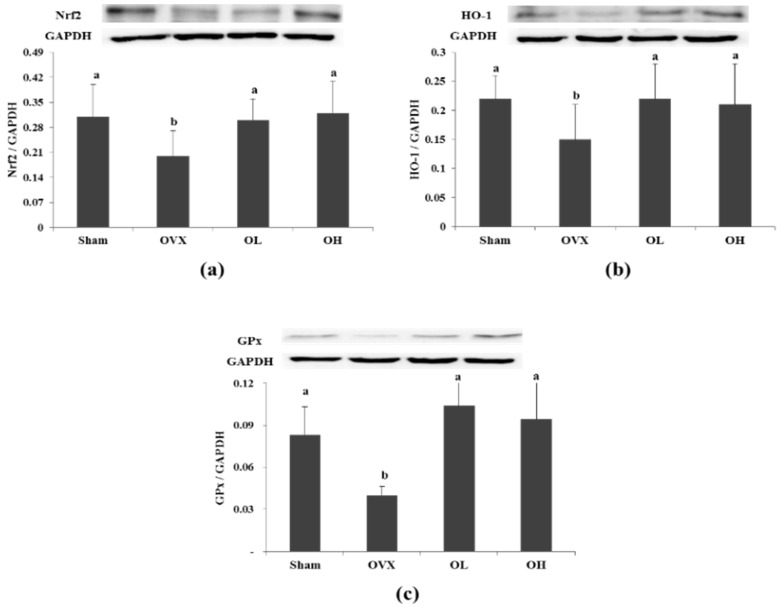
Protein expression of Nrf2, HO-1, and GPx in liver of each group. (**a**) Nrf2; (**b**) HO-1; (**c**) GPx. Values are mean ± SD (*n* = 7 for each group); the different letters (a > b > c) within a column indicate significant differences (*p* < 0.05) determined by Duncan’s multiple range test; Sham, the non-OVX control; OVX, the OVX-control; OL, the OVX + 100 mg/kg b.w. of hawthorn extract; OH, the OVX + 200 mg/kg b.w. of hawthorn extract; Nrf2, Nuclear factor erythroid 2–related factor; HO-1, Heme oxygenase-1; GPx, Glutathione peroxidase; GAPDH, Glyceraldehyde-3-phosphate dehydrogenase.

To examine whether the antioxidant defense system was modulated by hawthorn extract, Western blot analysis was performed. As shown in Figure 7, the protein levels of Nrf2 were lower following ovariectomy (*p* = 0.0204). However, the oral administration of hawthorn extract (100 and 200 mg/kg) reverses the suppression of Nrf2 protein levels (*p* = 0.0135 and *p* = 0.0202, respectively). The protein expression of HO-1 was lower in the OVX group than the Sham group (*p* = 0.0125), but significantly higher in the hawthorn-treated group than the OVX group (*p* < 0.05). The protein expression of GPx was lower in the OVX group compared with the Sham group (*p* = 0.001). However, hawthorn extract enhanced the protein expression of GPx (*p* = 0.05 and *p* = 0.003, respectively).

## 4. Discussion

Phenolic compounds extracted from fruit can prevent oxidative stress related to cardiovascular disease because of their antioxidant properties. Biological studies have reported that hawthorn has antioxidant properties that can quench free radicals and inhibit lipid peroxidation [30,31]. Because the components of hawthorn depend on a series of factors, such as cultivar, fertilization, maturation of the berries, harvest date, or habitat/location [15,32,33], we previously measured the phenol (1828.3 ± 58.4 mg GAE/g, 1948.9 ± 58.1 mg GAE/g, respectively), flavonoids (706.2 ± 135 mg RE/g, 1315.7 ± 86.9 mg RE/g, respectively), and DPPH (79.6% ± 1.3%, 83.6% ± 0.3%, respectively) levels using the same hawthorn extract used in the current study. The purpose of this study was to demonstrate the beneficial effects of hawthorn fruit extract in a mimic model for menopause. Our results showed that OVX rats presented elevated total cholesterol, LDL cholesterol, and non-HDL cholesterol levels in the serum and MDA levels in the liver as well as the reduced expression of Nrf2, HO-1, and GPx in the liver. Hawthorn extract treatment decreased the serum total cholesterol, LDL cholesterol, and non-HDL cholesterol levels in the OVX rats. Moreover, hawthorn extract was able to restrain the increase in body weight and uterine fat accumulation induced by OVX. Another important finding is that hawthorn extract increased the serum GPx activity and the expression of Nrf2, HO-1, and GPx in the liver of OVX rats.

To the best of our knowledge, this is the first study to examine the effects of hawthorn fruit extract administration on the lipid profiles and antioxidant defense system in OVX rats, and this is the first study showing that hawthorn, via the Nrf2/ARE-mediated pathway, increases antioxidant enzymes in the liver of OVX rats.

Ovariectomy in mice and rats is an experimental model used in many menopause studies. This procedure makes the acquisition of female rats without ovarian hormone secretion possible in a short period of time. In addition, ovariectomized rats show a high risk of osteoporosis symptoms [34], important cardiovascular dysfunctions [35], and uterine atrophy [36], as well as an imbalance between free radical production and antioxidant defense levels.

Body weight did not differ between the Sham and OVX groups at the beginning of the study, but eight weeks after ovariectomy, the body weight was higher in the OVX group by 1.36-fold, than the Sham group (*p* < 0.0001). The increase in body weight is considered a result of altered energy metabolism caused by estrogen deficiency favoring fat deposition [37]. Contrary to the increase in uterine fat weights, the uterine weights were found to be decreased in the OVX group. Such a decrease in uterine weights is a direct consequence of estrogen deficiency, which is required for the normal functioning and maintenance of the uterus [38]. Ryou *et al.* reported that the FER of OVX rats was far higher than that of normal female rats [39], and our study also showed a significant increase in OVX rats. Estrogen directly and indirectly modulates the activity of the molecules involved in orexigenic action, which induces an increase in food intake [40], but in this study, there was no difference in food intake in all OVX groups, which is in agreement with the observations of OVX rats in previous reports [41,42]. Our results demonstrated that the increase in body weight was not due to an increase in food intake. The administration of hawthorn extract did not affect food intake, but suppressed weight gain, FER, and uterine fat weight and increased the uterine weight in OVX rats. The antiobesity effects of hawthorn extract may be associated with its action in controlling fat synthesis and utilization. Hawthorn polyphenol extract suppresses the fatty acid synthetase gene in breast cancer cells [43] and stimulates fat oxidation through the activation of peroxisome proliferator–activated receptor alpha (PPAR α) in hamsters fed a high-fat diet [44].

The success of ovariectomy was also confirmed by examining the serum estradiol level. The serum estradiol levels were markedly decreased in all OVX groups and provided evidence of the success of the ovariectomy. Increases in plasma total cholesterol, LDL cholesterol, and triglycerides, and decreases in HDL cholesterol are common metabolic symptoms in menopausal woman [45]. In this study, the OVX group showed an increase in serum total cholesterol, LDL cholesterol, and non-HDL cholesterol levels, but no alterations in total triglycerides and HDL cholesterol levels. Hawthorn extract treatment significantly reduced the serum total cholesterol, LDL cholesterol, and non-HDL cholesterol levels in OVX rats. Previous studies have shown that hawthorn prevents coronary heart disease and hypercholesterolemia. Zhang *et al.* [46] demonstrated that supplementation with a 2% hawthorn powder diet for 12 weeks can lower serum total cholesterol and TG levels in a hypercholesterolemic rabbit model. The cholesterol-lowering effect of hawthorn may be associated with its regulation of bile acid synthesis or up-regulation of cholesterol 7 alpha-hydroxylase (CYP7A1) in the hypercholesterolemic rat model [47]. These results indicate that hawthorn extract has a protective effect on OVX rats by improving cholesterol levels. Additionally, AI was significantly increased in the OVX group, but both of the hawthorn extract groups were significantly decreased (by 17% and 26%, respectively). Postmenopausal changes in lipid metabolism may contribute to the increased risk of cardiovascular disease [2]. These findings suggest that hawthorn treatment effectively prevents estrogen deficiency–induced cardiovascular disease. 

Oxidative stress, a disparity between the rates of free radical production and elimination, occurs when the antioxidant mechanisms are overwhelmed. There is evidence that an oxidative imbalance occurs in women after menopause [48]. Ovariectomy may induce variations in antioxidant/oxidant status that can be detected in rat serum and liver [49]. The effect of OVX on hepatic CAT activity is contradictory. Kankofer *et al.* [49] demonstrated that CAT activity tends to be high in OVX animals, but Oztekin *et al.* [50] showed that OVX reduces the activity of this enzyme. However, in our study the activity of CAT in OVX rats was almost the same compared with the Sham group. The highest serum GPx activity was observed in rats in the OH group. Ha *et al.* [51] showed a reduction in GPx activity as a consequence of OVX-induced oxidative stress. Nevertheless, in this study, no significant changes were observed in serum GPx activity between the OVX and Sham groups. The mRNA and protein expression of GPx was found to be decreased in the liver of OVX rats, which might be due to the increased production of ROS and oxidative stress. Hawthorn can scavenge the superoxide radical, thus protecting GPx against inactivation [20,52]. These may be possible reasons for the increased mRNA and protein expression of GPx in OVX rats administrated hawthorn extract. These results appear to indicate a better protective effect of hawthorn extract against oxidative stress in the liver of OVX rats.

MDA is a product of the peroxidation of lipids and is often used to indicate the extent of oxidative stress. Ovariectomy-induced ROS accelerates the activity of lipid peroxidation in the polyunsaturated fatty acids of the cell membrane, and the increased level of MDA inversely influences the activity of antioxidant enzymes [53]. Ha *et al.* [51] and Topçuoglu *et al.* [54] reported that MDA levels in the liver were increased in OVX rats compared with the control group. Our result showed that hepatic MDA increased gradually following the ovariectomy in the OVX group, indicating that the deficiency of estrogen leads to increased oxidative stress. The administration of hawthorn extract improved the antioxidant defense system and decreased lipid peroxidation in the liver of OVX rats. 

It has been reported that the marked reduction of estrogen during menopause increases FFA levels [55]. This makes postmenopausal women more susceptible to metabolic syndrome and insulin resistance, both of which are implicated as risk factors for cardiovascular disease [56]. In the liver, estrogen deficiency results in the accumulation of TG in the cytosol of hepatocytes, which is a characteristic of nonalcoholic fatty liver disease (NAFLD) [57]. It has been well documented that increased hepatic fat accumulation is strongly associated with high circulatory FFA levels and increased intra-abdominal fat deposition [58]. In our study, there was no difference in serum FFA and hepatic TG levels between the OVX and Sham groups, which is consistent with the observations in previous studies [59,60]. These differences could depend on factors such as age, diet or body composition of the animals receiving the treatment, as well as the time of experimental period. However, the hawthorn extract–treated groups showed significantly decreased hepatic TG levels and the high dose of the hawthorn extract group showed significantly decreased serum FFA levels compared with the OVX group. Our results suggested that treatment with hawthorn extract inhibited hepatic TG accumulation through a decrease of serum FFA level.

The triglyceride accumulation in the liver is also known to be associated with oxidative stress. Nrf2 is the transcription factor that plays a key role in the activation of cellular responses to oxidative stress [61]. The mRNA expression of Nrf2 was markedly decreased in OVX rats, and the administration of hawthorn extract reverses the suppression. The protein expression was also decreased in rats in the OVX group. It has been documented that when Nrf2 levels are high, the *de novo* synthesized Nrf2 translocates to the nucleus and not only activates ARE gene expression, but also autoregulates its own gene expression [62]. Previous studies have reported that HO-1, the downstream gene of the Nrf2 pathway, is able to reduce oxidative injury, attenuate the inflammatory response, inhibit cell apoptosis, and maintain cellular homeostasis [63]. The protective effect of HO-1 is due to its catalysis of heme and the subsequent production of bioactive metabolites. A variety of dietary antioxidants (e.g., curcumin, resveratrol, selenium, carnosol) have the ability to induce the expression of HO-1 and to promote its antioxidative function [64]. The mRNA and protein expression of HO-1 was consistent with the mRNA and protein expression of Nrf2. In addition, the changes in the mRNA and protein expression of GPx agreed with the changes in the Nrf2 pathway. Thus, our findings suggested that the protective effects of hawthorn extract on the OVX rats might be due to the activation of the Nrf2 signaling pathway. This result is in accordance with the results of previous studies, which suggest that physiologically active components in hawthorn such as Procyanidin B2 might act as a mechanism for lowering oxidative stress [65]. Procyanidin B2 possesses strong free radical scavenging activity and antioxidant activity [66], and it has been demonstrated that Procyanidin B2 protects human colonic cells against oxidative stress through activation of the transcription factor Nrf2 via regulation of extracellular signal-regulated kinases (ERKs) and p38 signaling [67]. The data suggest that the protective action of Procyanidin B2 in hawthorn is related to the induction of antioxidant defense. Hawthorn extract proved its antioxidant ability through the up-regulation of Nrf2/HO-1, hence preserving GPx as well as reducing MDA levels in liver. 

There are limitations of this study that need to be addressed. First, estrogen deficiency also impairs glucose metabolism–related oxidative stress. Estrogen prevents disturbances in glucose metabolism by increasing insulin resistance and decreasing β-cell function and mass in OVX rats [68]. However, this study mainly focused on estrogen deficiency–induced oxidative stress, and further research should be performed to confirm the effect of hawthorn on insulin resistance. Second, the current study was conducted with only two doses of hawthorn administration and most of the parameters were not affected in a dose-dependent manner. However, it has been reported that the consumption of foods rich in antioxidants may be helpful in enhancing the beneficial effects of pharmacotherapy for postmenopausal patients [69]. Hawthorn might be a strong candidate because of its powerful antioxidative capacity.

## 5. Conclusions

In conclusion, the findings of this study add to the current knowledge about the effects of hawthorn extract on ovariectomy-induced dyslipidemia and oxidative stress, particularly regarding serum GPx activity, the hepatic MDA level, hepatic mRNA expression of Nrf2, HO-1, and GPx and protein expression of Nrf2, HO-1, and GPx. These results provide clinically relevant insight into the supplementation of hawthorn extract to prevent oxidative stress–related cardiovascular disease caused by estrogen deficiency. 

## Figures and Tables

**Table 1:** RT-PCR primer sequences.

Genes	Molecule Sequence (5′-3′)	Annealing	Cynyle
Nrf2			
Forward	CCATTTACGGAGACCCAC	55 °C	30
Reverse	TGAGCGGCAACTTTATTC		
HO-1			
Forward	TGCTCGCATGAACACTCTG	60 °C	45
Reverse	TCCTCTGTCAGCAGTGCCT		
GPx			
Forward	CTCTCCGCGGTGGCACAGT	58 °C	30
Reverse	CCACCACCGGGTCGGACATAC		
CAT			
Forward	GCGAATGGAGAGGCAGTGTAC	55.5 °C	30
Reverse	GAGTGACGTTGTCTTCATTAGCACTG		
GAPDH			
Forward	CCTCTCTCTTGCTCTCAGTAT	56 °C	33
Reverse	GTATCCGTTGTGGATCTGACA		

Nrf2, the nuclear factor erythroid 2–related factor; HO-1, Heme oxygenase-1; GPx, Glutathione peroxidase; CAT, Catalase; GAPDH, Glyceraldehyde-3-phosphate dehydrogenase; RT-PCR, reverse transcription-polymerase chain reaction.

**Table 2:** Food intake, food efficiency ratio, body weight, and organ weight.

	Sham	OVX	OL	OH
Food intake (g/day)	14.44 ± 3.73 ^ns^	13.68 ± 0.32	13.78 ± 0.90	12.67 ± 0.93
FER	0.08 ± 0.02 ^c^	0.23 ± 0.03 ^a^	0.16 ± 0.08 ^b^	0.13 ± 0.06 ^b,c^
Body weight (g)				
Initial	210.49 ± 5.79 ^ns^	212.19 ± 7.12	209.12 ± 14.84	214.46 ± 6.98
Final	264.87 ± 9.95 ^c^	359.63 ± 19.57 ^a^	309.37 ± 41.19 ^b^	291.48 ± 36.65 ^b,c^
Organ weight (g)				
Liver	7.21 ± 0.60 ^b^	8.80 ± 0.95 ^a^	7.69 ± 0.86 ^b^	7.16 ± 1.06 ^b^
Uterus	0.54 ± 0.09 ^a^	0.13 ± 0.05 ^b^	0.33 ± 0.23 ^a,b^	0.41 ± 0.33 ^a^
Uterine fat	3.40 ± 1.16 ^b^	7.37 ± 2.26 ^a^	5.25 ± 1.93 ^b^	5.04 ± 1.53 ^b^

FER, Food efficiency ratio; ^ns^, not significant; Values are mean ± SD. (*n* = 7 for each group). The different letters (a > b > c) within a column indicate significant differences (*p* < 0.05) determined by Duncan’s multiple range test; Sham, the non-OVX control; OVX, the OVX-control; OL, the OVX + 100 mg/kg b.w. of hawthorn extract; OH, the OVX + 200 mg/kg b.w. of hawthorn extract.

## Abbreviations

The following abbreviations are used in this manuscript:

LDL: low-density lipoprotein
VLDL: very low-density lipoprotein
HDL: high-density lipoprotein
ROS: reactive oxygen species
CAT: catalase
SOD: superoxide dismutase
GPx: glutathione peroxidase
Nrf2: nuclear factor erythroid 2 related factor
HO-1: heme oxygenase-1
FFA: free fatty acids
GST: glutathione S-transferase
ARE: antioxidant response element
AI: Atherogenic Index
TG: triglyceride
MDA: malondialdehyde
TBA: thiobarbituric acid
TCA: trichloroacetic acid
RT-PCR: reverse transcription polymerase chain reaction
GAPDH: Glyceraldehyde-3-phosphate dehydrogenase
FER: food efficiency ratio
NAFLD: nonalcoholic fatty liver disease
ERKs: extracellular signal-regulated kinases
OVX: ovariectomized
PPAR α: peroxisome proliferator-activated receptor alpha
CYP7A1: cholesterol 7alpha-hydroxylase
